# Switching from anti-CD20 therapies to cladribine and vice versa – Analysis of a German relapsing multiple sclerosis cohort

**DOI:** 10.1016/j.neurot.2025.e00812

**Published:** 2025-12-04

**Authors:** Franz Felix Konen, Steffen Pfeuffer, Konstantin Fritz Jendretzky, Klaus Gehring, Birte Elias-Hamp, Kurt-Wolfram Sühs, Stephan Halle, Korbinian Brand, Ralf Lichtinghagen, Eline Willemse, Marc Pawlitzki, Jens Kuhle, Sven G. Meuth, Christoph Kleinschnitz, Refik Pul, Thomas Skripuletz

**Affiliations:** a Department of Neurology, Hannover Medical School, Carl-Neuberg-Str. 1, 30625 Hannover, Germany; b Department of Neurology, University Hospital of Giessen and Marburg, Justus-Liebig-University-Giessen, Klinikstr. 33, 35392 Giessen, Germany; c Neurozentrum am Klosterforst, Hanseaten-Platz 1, 25524 Itzehoe, Germany; d Praxis Dr. Elias-Hamp, Bengelsdorfstr. 5, 22179 Hamburg, Germany; e Institute of Clinical Chemistry and Laboratory Medicine, Hannover Medical School, Carl-Neuberg-Str. 1, 30625 Hannover, Germany; f Institute of Immunology, Hannover Medical School, Carl-Neuberg-Str. 1, 30625 Hannover, Germany; g Multiple Sclerosis Centre and Research Center for Clinical Neuroimmunology and Neuroscience (RC2NB), Neurology, Departments of Biomedicine and Clinical Research, University Hospital and University of Basel, Basel, Switzerland; h Department of Neurology, University Hospital and University of Basel, Spitalstrasse 2, CH-4031 Basel, Switzerland; i Department of Neurology, Medical Faculty, Heinrich-Heine-University Düsseldorf, Duesseldorf, Germany; j University Medicine Essen, Neurology, Essen, Germany; k Center for Translational Neuro- and Behavioral Sciences, University Medicine Essen, Neurology, Essen, Germany

**Keywords:** Active multiple sclerosis, Anti-CD20-Therapy, Cladribine, Switch

## Abstract

Anti-CD20 antibodies and cladribine are established therapies for active relapsing multiple sclerosis (RMS). Increasing evidence suggests that switching between these therapies may be beneficial in patients with ongoing disease activity under current treatment. In this multicenter retrospective study across six German MS centres, a total of 90 patients with active RMS were considered for inclusion, of whom 71 patients were switched either from anti-CD20 antibodies to cladribine (n ​= ​31) or from cladribine to anti-CD20 antibodies (n ​= ​40), with a minimum follow-up of 12 months. At treatment initiation, patients switching from anti-CD20 antibodies were older, had a longer disease duration, and a higher disability score compared to those switching from cladribine (p ​= ​0.0040, p ​= ​0.0447, p ​= ​0.0028, respectively). The primary reason for switching was disease activity. Following the switch, the proportion of patients with relapsing disease activity was markedly reduced (from 55 ​% to 16 ​% for anti-CD20 to cladribine, and from 83 ​% to 25 ​% for cladribine to anti-CD20). Clinical outcomes improved, while serum biomarkers such as neurofilament light chain and glial fibrillary acidic protein remained stable over six months. Notably, the prevalence of hypogammaglobulinemia decreased after switching from anti-CD20 therapies to cladribine. These results indicate that patients with active RMS can achieve clinical stabilization after switching therapies in either direction, underscoring the complementary mechanisms of action and the safety of such an approach in real-world practice.

## Introduction

Relapsing multiple sclerosis (RMS) is the most common neuroimmunological disease in young adults and might lead to accumulation of neurological disability in the disease course [1]. To avoid disability accrual, effective immunomodulatory treatment is the most important factor to be considered [1,2]. If disease activity still occurs, a switch to a more effective disease-modifying therapy (DMT), such as anti-CD20 therapies or cladribine, is recommended [3]. Cladribine and anti-CD20 antibodies are effective therapies approved for people with highly active RMS [[4], [5], [6]]. Both therapies involve depletion of B cells in their mode of action, which occurs continuously under treatment with anti-CD20 antibodies and transient for most B cell subsets except memory B cells in case of immune reconstitution therapy with cladribine [7,8]. Although these therapies are effective in many patients, some still exhibit disease activity, making an alternative treatment necessary [9,10]. Recent data indicate that patients showing signs of chronic or acute inflammatory disease activity under anti-CD20 therapies may benefit from a switch to cladribine and vice versa [[11], [12], [13]].

As a consequence of continuous B cell depletion during prolonged treatment with anti-CD20 antibodies such as ocrelizumab, an increased occurrence of infections and hypogammaglobulinemia has been observed [14]. Coupled with immunosenescence, the risk of infections is particularly increased in the elder population [15]. Especially in this older patient population, cladribine represent a viable treatment option and a potential “exit therapy,” as immune reconstitution therapy with cladribine is rarely associated with hypogammaglobulinemia [8,16]. In a recent pilot study, 14 patients were switched from anti-CD20 antibodies to cladribine with promising results in terms of disease control and laboratory parameters [17]. A switch may also become necessary during treatment with cladribine, due to either recurring disease activity or lymphopenia [18]. For these patients, switching to anti-CD20 antibody therapies represents a reasonable strategy. Whereas some real-world studies have reported switching cladribine-treated patients to anti-CD20 therapies, a systematic investigation of the cladribine/anti-CD20 treatment sequence based on a larger cohort has been lacking to date.

Thus, the objective of the present study was to evaluate the effectiveness and safety of switching patients with active RMS from anti-CD20 antibody therapies to cladribine and vice versa under real-world conditions.

## Methods

### Study design and patients

Six ambulatory German MS centers provided the patient pool for this data analysis. Data were retrieved retrospectively from patient records. Adults with highly active RMS, who were treated with anti-CD20 antibodies and switched to cladribine or vice versa were included in the analysis, if 12-month follow-up data were available after switching. There was no pre-planned schedule, included patients underwent clinical evaluations at least semi-annually, with semi-annual to annual 1.5 or 3T MRI scans in accordance with the MAGNIMS-CMSC-NAIMS guidelines to detect new T2-hyperintense lesions or gadolinium-enhancing lesions [19]. Necessity and timing of the switch as well as the subsequent therapy were determined by the treating physician.

### Outcomes

Clinical and paraclinical outcome parameters included relapse activity, expanded disability status scale (EDSS) score, MRI progression, and no evidence of disease activity-3 (NEDA-3) [[20], [21], [22]]. NEDA-3 was defined as no relapses, no progression independent of relapse activity (PIRA), and no isolated MRI activity [[20], [21], [22], [23]]. Relapses were defined as no onset or exacerbation of neurological symptoms for at least 24 ​h and required an increase of at least one function score of the EDSS. MRI progression was defined as new T2-lesions, gadolinium-enhancing lesions, or enlarging lesions [19,23]. Disability worsening was considered if EDSS increased as follows: +1.5 points (baseline: 0); +1.0 point (baseline: 1–5.5); +0.5 points (baseline: ≥6.0) and this required confirmation six months later [22]. PIRA was assessed as applying the abovementioned EDSS steps [22].

Laboratory parameters included serum levels of immunoglobulin G (IgG), neurofilament light chain (sNfL), and glial fibrillary acidic protein (sGFAP) [[24], [25], [26]]. IgG concentrations were measured in the accredited labs at the respective study site following international standards, defining a hypogammaglobulinemia as serum concentrations below 7 ​g/l [26]. Patients with anti-CD20 therapies were switched due to hypogammaglobulinemia, when IgG levels were distinctly reduced below 7 ​g/l. Lymphocyte counts were obtained before each treatment was initiated from the accredited labs at the respective study site following international standards [27]. Patients with cladribine therapy were switched when lymphopenia was moderate (0.5–0.8 ​× ​10^3^/μl) to severe (0.2–0.5 ​× ​10^3^/μl) and/or persistent. sNfL and sGFAP were measured before the second treatment was initiated and 6 months after the switch. sNfL was measured using the Elecsys NfL RUO assay and sGFAP was measured using the Elecsys GFAP RUO (research use only) assay (both Roche Diagnostics Deutschland GmbH, Mannheim, Germany) on a cobas e801 analysis system according to the manufacturer's guidelines. sNfL and sGFAP Z scores normalized for age, sex (only sGFAP) and body mass index were calculated as described before [28,29].

### Statistical analysis

Descriptive statistical analyses were performed on the total population and subgroups stratified by reason for the switch (disease activity, safety issues) and by age (<45 vs. ≥45 years). Age was dichotomized to maintain comparable group sizes while achieving the highest possible age threshold for the older group. D'Agostino-Pearson-test was used to assess for parametrical distribution of decimal variables. Parametrical data were described as mean with standard deviation, whereas non-parametrical data were described as median with interquartile range (IQR). Group comparison was achieved via the Wilcoxon Rank sum Test for decimal data and via Chi2 test for binary data. For analysis of paired values, either the paired *t*-test (parametrically distributed values) or the Wilcoxon matched pairs test (nonparametrically distributed values) were used. For comparison of more than two groups, the post hoc test for multiple comparison error (Bonferroni correction) was performed. All tests were two-sided and significance was declared at the 0.05 level. No formal sample size calculation was conducted due to the retrospective observational character of the study. The sample size depended on the number of patients having 12-month follow-up data after switching treatment. Statistical analysis of all collected data was performed using Graphpad Prism (La Jolla, CA, USA; version 8.4.3) as well as SPSS 29.0 and its respective extension packages (IBM Co., Armonk, New York, USA).

### Standard protocol approvals, registrations, and patient consents

The investigation was approved by the institutional ethics committees and conducted in accordance with the Declaration of Helsinki (1964) and its later amendments. Due to the retrospective study approach and the usage of medical record data only, the need for written consent for participation and publication was waived by the institutional ethics committees. In a subcohort of patients, serum samples were available for the analysis of sNfL and sGFAP. These patients provided written informed consent for the use of their samples, study participation and publication. This study adhered to the Strengthening the Reporting of Observational Studies in Epidemiology (STROBE) reporting guideline.

### Data availability

The datasets generated and/or analysed during the current study are available from the corresponding author on reasonable request from qualified investigators.

## Results

### Patients

A total of 90 patients was considered suitable for the present study, with 48 patients receiving anti-CD20 antibodies (ocrelizumab, n ​= ​37; ofatumumab, n ​= ​11) switching to cladribine and 42 switching from cladribine to anti-CD20 antibodies (ocrelizumab, n ​= ​25; ofatumumab, n ​= ​16; ublituximab, n ​= ​1). Follow-up data over a minimum of 12 months were available from 31 patients switching to cladribine (ocrelizumab, n = 28; ofatumumab, n = 3; referred to as anti-CD20/CladT group) and 40 patients switching to anti-CD20 antibodies (ocrelizumab, n = 24; ofatumumab, n = 16; referred to as CladT/anti-CD20 group). Baseline characteristics are depicted in Table 1.

**Table 1:** Baseline characteristics before initiation of the first treatment.

	First treatment		
	Anti-CD20-antibodies (N ​= ​31)	Cladribine (N ​= ​40)	p-value
Age [years], median (IQR)	44 (34–53)	37.5 (27–42)	**0.0040**
Females, n (%)	22 (71 ​%)	29 (73 ​%)	0.9999
EDSS, median (IQR)	4.0 (3–6)	2.5 (2–3.5)	**0.0028**
Number of previous therapies, median number (IQR)	3 (2–5)	2 (2–4)	0.3814
Therapy naïve at first therapy initiation, n (%)	4 (13 ​%)	10 (25 ​%)	0.2423
Months from MS diagnosis to first therapy, median (IQR)	72 (21–193)	46 (7–106)	**0.0447**
Treatment courses first therapy, median (IQR)^a^	4 (2–5)	2 (2–4)	Not comparable
Days between therapies, median (IQR)	213 (159–291)	521 (365–886)	Not comparable
Reasons for treatment switch			
Relapse	12 (39 ​%)	19 (47.5 ​%)	0.6337
PIRA	5 (16 ​%)	1 (2.5 ​%)	0.0794
MRI activity	5 (16 ​%)	14 (35.0 ​%)	0.1057
Adverse drug reactions	9 (29 ​%)	6 (15.0 ​%)	0.2406
Lymphopenia	0	6 (15.0 ​%)	

EDSS ​= ​Expanded Disability Status Scale; IQR ​= ​interquartile range; PIRA ​= ​progression independent of relapse activity.

a For cladribine: one course ​= ​1 treatment week; for ocrelizumab: one course ​= ​600 ​mg.

Patients receiving anti-CD20 antibodies as first therapy were significantly older (median age 44 vs. 37.5 years, p ​= ​0.0040), had a longer disease course before treatment initiation (72 vs. 46 months, p ​= ​0.0447), and had a higher EDSS score (median 4.0 vs. 2.5, p ​= ​0.0028) than patients receiving cladribine. After switching, the median follow-up duration was 693 days (IQR: 415–861) for the anti-CD20/CladT group and 720 days (IQR: 383–1080) for the CladT/anti-CD20 group.

The main reason for switching was disease activity (71 ​% in anti-CD20/CladT group and 85 ​% in the CladT/anti-CD20 group) with the highest prevalence of relapsing disease activity under treatment. Comparing both groups, onset of relapses, PIRA and disease activity in MRI was not statistically different. Adverse events as reason for switch were lymphopenia in all patients with initial treatment with cladribine and hypogammaglobulinemia (67 ​%), liver injury (22 ​%) and neutropenia (11 ​%) in all patients with initial treatment with anti-CD20-therapies. In patients switching due to lymphopenia, 3/6 revealed moderate and severe lymphopenia under cladribine treatment each. In patients switching due to hypogammaglobulinemia, IgG levels ranged from 4.3 ​g/l to 6.7 ​g/l.

### Effectiveness outcomes

**Fig. 1:**
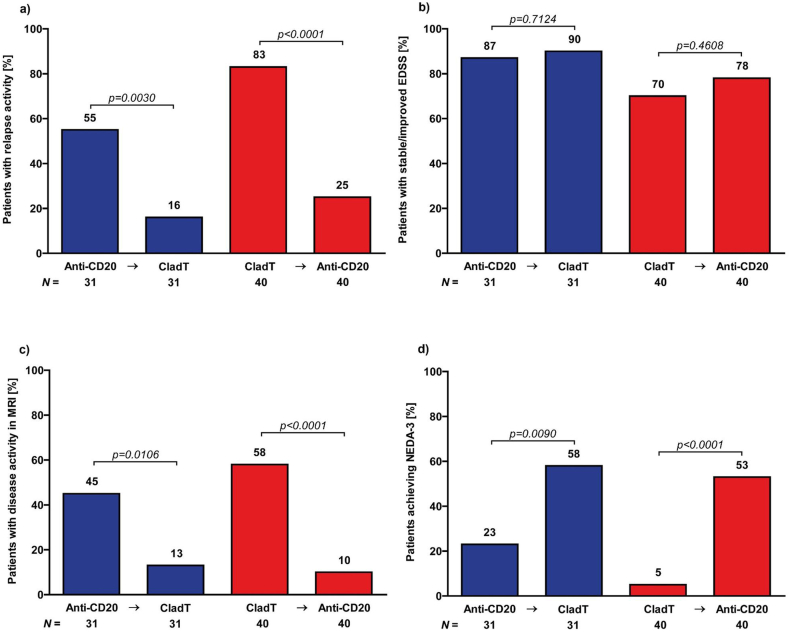
Effectiveness outcomes after switching from anti-CD20-antibodies to cladribine and vice versa Proportion of patients with relapsing disease activity (a), stable or improved EDSS (b), disease activity in MRI (c), and achieving NEDA-3 (d) before and after treatment switch. Blue columns represent the anti-CD20-therapy to cladribine switching patients and red columns the cladribine to anti-CD20-therapy switching patients. Sample size shown per group. EDSS ​= ​Expanded Disability Status Scale; MRI ​= ​magnetic resonance imaging; NEDA-3 ​= ​no evidence of disease activity (no relapses, no PIRA (progression independent of relapse activity), no isolated MRI activity).

Following the switch, a reduction was observed in the proportion of patients with relapsing activity (anti-CD20/CladT group: from 55 ​% to 16 ​%; CladT/anti-CD20 group: from 83 ​% to 25 ​%; Fig. 1a). In patients with first therapy of cladribin and switch to anti-CD20 therapies, relapses occurred after a median time of 16 months (IQR: 6–35) under cladribine and after 15 (IQR: 5–35) under anti-CD20 therapies with a median time from relapse under cladribine to switch of two months (IQR: 2–4). In patients with first therapy of anti-CD20 therapies and switch to cladribine, relapses occurred after a median time of 11 months (IQR: 7–18) under anti-CD20 therapies and after 6 (IQR: 3–10) under cladribine with a median time from relapse under anti-CD20 therapies to switch of four months (IQR: 1–6). The proportion of patients with stable or improved EDSS increased slightly during the follow-up after switch (anti-CD20/CladT group: from 87 ​% to 90 ​%; CladT/anti-CD20 group: from 70 ​% to 78 ​%; Fig. 1b). A decrease in disease activity on MRI was also noted (anti-CD20/CladT group: from 45 ​% to 13 ​%; CladT/anti-CD20 group: from 58 ​% to 10 ​%; Fig. 1c). Consequently, the proportion of patients achieving NEDA-3 increased from 23 ​% to 58 ​% in the anti-CD20/CladT group and from 5 ​% to 53 ​% in the CladT/anti-CD20 group (Fig. 1d).

### Laboratory results

**Fig. 2:**
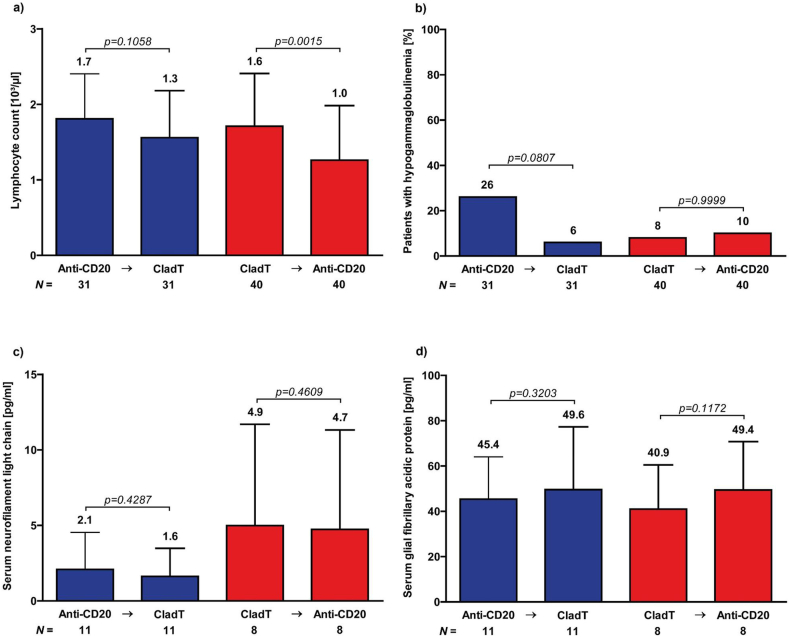
Laboratory analyses before and after switch Mean absolute lymphocyte count and standard deviation (SD) before treatment administration (a), proportion of patients with hypogammaglobulinemia under therapy (b), mean neurofilament light chain (c) and glial fibrillary acidic protein (d) concentrations and SD before administration of the second treatment and 6 months after switch. Blue columns represent the anti-CD20-therapy to cladribine switching patients and red columns the cladribine to anti-CD20-therapy switching patients. Sample size shown per group.

Sufficient lymphocyte recovery was observed before administration of each treatment (mean lymphocyte count (standard deviation, SD): anti-CD20/CladT 1.7 (0.6) and 1.3 (0.6); CladT/anti-CD20 1.6 (0.5) and 1.0 (0.4); Fig. 2a). The prevalence of hypogammaglobulinemia was lower under cladribine after the switch from anti-CD20 therapies (from 26 ​% to 6 ​% for anti-CD20/CladT; from 8 ​% to 10 ​% for anti-CD20/CladT; Fig. 2b). Mean absolute sNfL (anti-CD20/CladT (SD): from 2.1 ​pg/ml (1.4 ​pg/ml) to 1.6 ​pg/ml (1.1 ​pg/ml), p ​= ​0.4287; CladT/anti-CD20 (SD): from 4.9 ​pg/ml (3.1 ​pg/ml) to 4.7 ​pg/ml (3.0 ​pg/ml); Fig. 2c) and sGFAP (anti-CD20/CladT (SD): from 45.4 ​pg/ml (19 ​pg/ml) to 49.6 ​pg/ml (28 ​pg/ml), p ​= ​0.3203; CladT/anti-CD20 (SD): from 40.9 ​pg/ml (20 ​pg/ml) to 49.4 ​pg/ml (21 ​pg/ml), p ​= ​0.1172; Fig. 2d) concentrations did not change significantly comparing values before and 6 months after the respective switch. sNfL concentrations at baseline (p ​= ​0.5357), after 6 months (p ​= ​0.3423), as well as sGFAP concentrations at baseline (p ​= ​0.3423) and after 6 months (p ​= ​0.7102) did not differ significantly between both groups. Similar results were found for Z scores of sNfL (anti-CD20/CladT (SD): from 0.3 (2.1) to 0.1 (1.9), p ​= ​0.9658; CladT/anti-CD20 (SD): from 1.2 (2.1) to 1.1 (2.1), p ​= ​0.9375) and sGFAP (anti-CD20/CladT (SD): from 0.5 (1.5) to 0.5 (1.5), p ​= ​0.7646; CladT/anti-CD20 (SD): from 0.4 (1.5) to 0.9 (1), p ​= ​0.0781). sNfL Z scores at baseline (p ​= ​0.3511), after 6 months (p ​= ​0.1232), as well as sGFAP concentrations at baseline (p ​= ​0.9678) and after 6 months (p ​= ​0.4068) did not differ between both groups. When absolute concentrations as well as Z scores for sNfL and sGFAP were adjusted for EDSS, time since last relapse, time since MS diagnosis, number of previous DMT, T2-lesion load in MRI and age (for absolute concentrations only), no differences were detected for any analysis.

### Subgroup analysis: switch due to disease activity vs. adverse events

As depicted in Table 1, most patients of both groups switched due to disease activity (anti-CD20/CladT: relapses 39 ​%, PIRA 16 ​%, disease activity in MRI 16 ​%; CladT/anti-CD20: relapses 47.5 ​%, PIRA 2.5 ​%, disease activity in MRI 35 ​%). Baseline characteristics were not significantly different between patients who switched because of disease activity or adverse events (Supplementary Table S1).

**Fig. 3:**
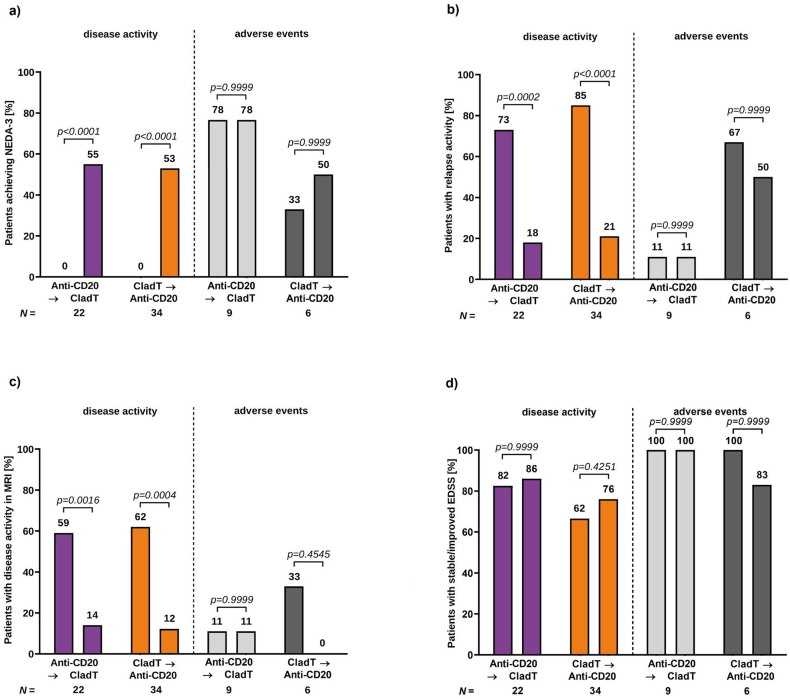
Effectiveness outcomes after switching from anti-CD20-antibodies to cladribine and vice versa in patients with switch due to disease activity and adverse events Proportion of patients achieving NEDA-3 (a), proportion of patients with relapsing disease activity (b), disease activity in MRI (c), and stable or improved EDSS (d) before and after treatment switch. Purple and light grey columns represent the anti-CD20-therapy to cladribine switching patients and orange and dark grey columns the cladribine to anti-CD20-therapy switching patients. Sample size shown per group. EDSS ​= ​Expanded Disability Status Scale; MRI ​= ​magnetic resonance imaging; NEDA-3 ​= ​no evidence of disease activity (no relapses, no PIRA (progression independent of relapse activity), no isolated MRI activity).

In terms of effectiveness, patients who switched due to disease activity experienced profound benefits from the switch. In this subgroup, the proportion of patients achieving NEDA-3 increased (0 ​%–55 ​% and 53 ​% for anti-CD20/CladT and CladT/anti-CD20, respectively; Fig. 3a), relapsing activity declined (anti-CD20/CladT: 73 ​%–18 ​%; CladT/anti-CD20: 85 ​%–21 ​%; Fig. 3b), and fewer patients experienced disease activity in MRI after the switch (anti-CD20/CladT: 59 ​%–14 ​%; CladT/anti-CD20: 62 ​%–12 ​%; Fig. 3c). The proportion of patients with stable or improved EDSS increased slightly after treatment switch (anti-CD20/CladT: 82 ​%–86 ​%; CladT/anti-CD20: 62 ​%–76 ​%; Fig. 3d).

Effectiveness outcomes remained stable in the small group of patients who switched from anti-CD20 to cladribine due to adverse events and vice versa (n ​= ​9 and n ​= ​6, Fig. 3a–d). In the group switching from cladribine to anti-CD20 due to adverse events, statistically non-significant improvements were observed in terms of NEDA-3 (anti-CD20/CladT: 78 ​%78 ​%; CladT/anti-CD20: 33 ​%–50 ​%; Fig. 3a), relapsing activity (anti-CD20/CladT: 11 ​%–11 ​%; CladT/anti-CD20: 67 ​%–50 ​%; Fig. 3b), and disease activity in MRI (anti-CD20/CladT: 11 ​%–11 ​%; CladT/anti-CD20: 33 ​%–0 ​%; Fig. 3c), whereas the proportion achieving EDSS improvement/stabilization non-significantly decreased (anti-CD20/CladT: 100 ​%–100 ​%; CladT/anti-CD20: 100 ​%–83 ​%) (Fig. 3d).

**Fig. 4:**
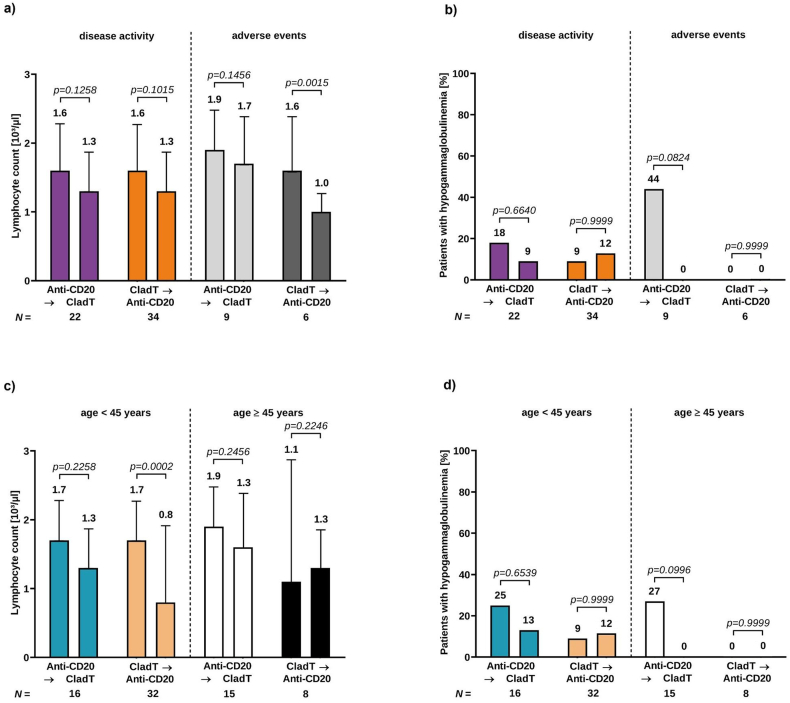
Stratification by reason for switch and age Stratification by reason for switch (a, b) and age (c, d); mean absolute lymphocyte count and standard deviation (SD) before treatment administration (a, c) and proportion of patients with hypogammaglobulinemia under therapy (b, d). In the analyses stratified by switch reason (disease activity, adverse events), purple and light grey columns represent the anti-CD20-therapy to cladribine switching patients and orange and dark grey columns the cladribine to anti-CD20-therapy switching patients. In the analyses stratified by age (<45 years, ≥45 years), light blue and white columns represent the anti-CD20-therapy to cladribine switching patients and light orange and black columns the cladribine to anti-CD20-therapy switching patients. Sample size shown per group. EDSS ​= ​Expanded Disability Status Scale; MRI ​= ​magnetic resonance imaging; NEDA-3 ​= ​no evidence of disease activity (no relapses, no PIRA (progression independent of relapse activity), no isolated MRI activity).

Mean lymphocyte counts remained within the normal range before administration of each treatment, independent of the reason documented for switching (switch due to disease activity (SD): anti-CD20/CladT 1.6 (0.8) and 1.3 (0.7); CladT/anti-CD20 1.6 (0.5) and 1.3 (0.4); switch due to adverse events: anti-CD20/CladT 1.9 (1.2) and 1.7 (1.0); CladT/anti-CD20 1.6 (0.9) and 1.0 (0.5); Fig. 4a). The proportion of patients with hypogammaglobulinemia was lower under cladribine therapy than under anti-CD20 antibodies (Fig. 4b: switch reason disease activity: anti-CD20/CladT 18 ​%–9 ​%, CladT/anti-CD20 9 ​%–12 ​%; switch reason adverse events: anti-CD20/CladT 44 ​%–0 ​%, CladT/anti-CD20 0 ​%-0 ​%). The difference was more similar in sub analysis considering reason for switch than age, although not reaching statistical significance (Fig. 4c; age <45 years: anti-CD20/CladT 25 ​%–13 ​%, CladT/anti-CD20 9 ​%–12 ​%; age ≥45 years: anti-CD20/CladT 27 ​%–0 ​%, CladT/anti-CD20 0 ​%–0 ​%).

### Subgroup analysis by age: <45 vs. ≥45 years

Among the 31 patients switching from anti-CD20 antibodies, 16 patients were <45 and 15 were ≥45 years of age at initiation of anti-CD20 treatment. Among the 40 patients switching from cladribine, 32 patients were <45 and 8 were ≥45 years of age at initiation of cladribine. Baseline characteristics were comparable between patients <45 and ​≥ ​45 years with the exception of the EDSS, which was significantly lower in younger patients of the group switching from cladribine to anti-CD20 therapies (2 vs. 4.5, p ​= ​0.0121; Supplementary Table S2).

**Fig. 5:**
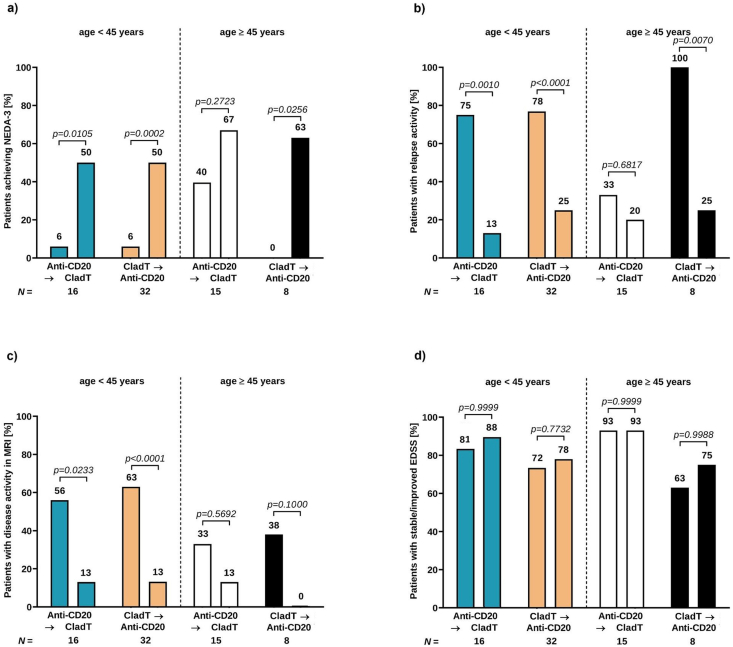
Effectiveness outcomes after switching from anti-CD20-antibodies to cladribine and vice versa in patients below and above the age of 45 years Proportion of patients achieving NEDA-3 (a), proportion of patients with relapsing disease activity (b), disease activity in MRI (c), and stable or improved EDSS (d) before and after treatment switch. Light blue and white columns represent the anti-CD20-therapy to cladribine switching patients and light orange and black columns the cladribine to anti-CD20-therapy switching patients. Sample size shown per group. EDSS ​= ​Expanded Disability Status Scale; MRI ​= ​magnetic resonance imaging; NEDA-3 ​= ​no evidence of disease activity (no relapses, no PIRA (progression independent of relapse activity), no isolated MRI activity).

In terms of effectiveness, beneficial effects were observed in both age groups, independent of the direction of the switch. The proportion of patients achieving NEDA-3 increased (age <45 years: anti-CD20/CladT from 6 ​% to 50 ​%, CladT/anti-CD20 from 6 ​% to 50 ​%; age ≥45 years: anti-CD20/CladT from 40 ​% to 67 ​%, CladT/anti-CD20 from 0 ​% to 63 ​%; Fig. 5a). Declining proportions were observed for relapsing activity (age <45 years: anti-CD20/CladT from 75 ​% to 13 ​%, CladT/anti-CD20 from 78 ​% to 25 ​%; age ≥45 years: anti-CD20/CladT from 33 ​% to 20 ​%, CladT/anti-CD20 from 100 ​% to 25 ​%; Fig. 5b) and disease activity in MRI (age <45 years: anti-CD20/CladT from 56 ​% to 13 ​%, CladT/anti-CD20 from 63 ​% to 16 ​%; age ≥45 years: anti-CD20/CladT from 33 ​% to 13 ​%, CladT/anti-CD20 from 38 ​% to 0 ​%; Fig. 5c). Concerning the proportion of patients with stable or improved EDSS, no significant change was observed in both age groups (age <45 years: anti-CD20/CladT from 81 ​% to 88 ​%, CladT/anti-CD20 from 72 ​% to 78 ​%; age ≥45 years: anti-CD20/CladT from 93 ​% to 93 ​%, CladT/anti-CD20 from 63 ​% to 75 ​%; Fig. 5d).

Independent of age, lymphocyte counts remained within the normal range for both directions of the switch (age <45 years: anti-CD20/CladT 1.7 (0.8) and 1.3 (0.7), p ​= ​0.2258; CladT/anti-CD20 1.7 (0.5) and 0.8 (0.4); age ≥45 years: anti-CD20/CladT 1.9 (1.2) and 1.3 (1.0); CladT/anti-CD20 1.1 (0.6) and 1.3 (0.8); Fig. 4c). Consistent with the overall population, the proportion of patients with hypogammaglobulinemia was lower under cladribine therapy than under anti-CD20 antibodies in both age groups, although not reaching statistical significance (Fig. 4: age <45 years: anti-CD20/CladT 25 ​%–13 ​%, CladT/anti-CD20 9 ​%–12 ​%; age ≥45 years: anti-CD20/CladT 27 ​%–0 ​%, CladT/anti-CD20 0 ​%–0 ​%).

## Discussion

This multicentric retrospective analysis evaluated the switch between anti-CD20 antibodies and cladribine in both directions. Overall, results demonstrated beneficial effects of the switch as reflected by improvements in several measures of effectiveness, including relapse activity, EDSS, MRI disease activity, and achievement of NEDA-3 status. Clinical and radiological improvements were further supported by stable levels of sNfL and gsGFAP, indicating no increase in subclinical disease activity.

Patients receiving anti-CD20 therapies as first treatment in this cohort had a higher amount of prior therapies and were more affected by disease as indicated by higher median EDSS scores (4.0 vs. 2.5). In spite of these less favourable conditions, a substantial proportion of patients achieved disease control after switching to cladribine. A decrease in relapse rates and MRI activity has been observed in patients switching from ocrelizumab to cladribine in previous studies, alleviating concerns about disease reactivation during de-escalation from monoclonal antibody therapy [11,17,23]. Due to its small molecular size and thus ability to establish significant concentrations in the cerebrospinal fluid, cladribine is supposed to affect pathological parameters of central inflammation, which may explain the observed additional benefits of switching [30,31]. Beyond the effects on B cells, cladribine also leads to a targeted temporary reduction of T cells and shows compartment-specific activity [8,[32], [33], [34], [35], [36]]. Since the more affected RMS patients in the present study were treated with anti-CD20 therapies in the first place, the therapeutic sequence of cladribine following anti-CD20 therapies seems to provide additional beneficial therapeutic effects. However, the potential contribution of overlapping immunological effects in the early phase after switching needs to be considered. On the one hand, residual activity of the previously administered DMT together with the newly initiated therapy could theoretically amplify immunosuppressive effects and thereby support the favourable clinical outcome observed after switching in both directions. On the other hand, the majority of patients (71 ​% in the anti-CD20/CladT group and 85 ​% in the CladT/anti-CD20 group) were switched due to ongoing disease activity, indicating insufficient efficacy or a lack of treatment response to the preceding DMT. This makes a causative influence of combined treatment mechanisms on the observed beneficial outcome less likely.

The optimal timing of the switch remains an important clinical question. Considering a median time to B-cell repletion after ocrelizumab discontinuation of 72 weeks (range 27–175 weeks) according to a long-term follow-up study, it is likely that residual effects of ocrelizumab were still present when cladribine was initiated [37]. A median time of 7 months was observed before patients were switched to cladribine, implying that cladribine was initiated at the time the next dose of ocrelizumab would have been due, which is in line with the recommendations of the German disease-oriented Competence Network Multiple Sclerosis (KKNMS) to wait 6–12 months after the last dose of ocrelizumab or ofatumumab to initiate cladribine [38]. Following a similar approach, a subset of patients from an Australian observational study received the first course of cladribine at the time of the next scheduled ocrelizumab dose and no safety signals were reported [12]. In one quart of patients cladribine was initiated earlier, within 5 months after the last anti-CD20 dose due to disease activity. In these patients, lymphocyte counts had recovered to normal ranges thus patients were able to switch at an earlier time point as indicated by disease activity, resembling the protocol from an US study, where patients were switched from ocrelizumab to cladribine after a median time of 155 days [11]. The feasibility of this is further supported by guidelines from the French Multiple Sclerosis Society that recommend a wash-out period of at least 3 months when switching from ocrelizumab [39]. Overall, prior data regarding the switch from anti-CD20 antibodies to cladribine in a pilot study with 14 patients have been confirmed in the larger cohort investigated here [17].

Our additional subgroup analysis of older patients (>45 years) suggests that switching from anti-CD20 therapies to cladribine may also be a viable option in this age group. This approach could help maintain disease control in active patients while minimizing long-term risks associated with persistent B-cell depletion and hypogammaglobulinemia, particularly in the context of immunosenescence [14,15].

Patients experiencing disease activity or persistent lymphopenia under cladribine could be effectively stabilised by anti-CD20-therapies such as ocrelizumab, ublituximab or ofatumumab. Switching these patients to another high efficacy therapy is in line with current expert opinions [40,41]. Furthermore, the benefits observed after the switch underscore that cladribine initiation does not represent an irreversible treatment decision, and transitioning to therapies such as anti-CD20 monoclonal antibodies remains a viable option.

In addition to the benefits observed from switching either way, most probably due to the complementarity in the mode of action of anti-CD20 therapies and cladribine, there were no severe safety signs observed. The switch was well tolerated and prior IgG deficiency resolved after switching to cladribine, which has been ascribed to the prompt recovery of B cells following the rapid decrease after cladribine initiation [8].

In the present study, further support for switching in both directions was provided by stable sNfL and sGFAP concentrations and Z scores over the 6 months following the switch [24,25]. Although most patients switched therapies due to disease activity, the last clinical relapse or MRI activity had occurred several weeks to months before the switch and subsequent blood sampling. Therefore, elevated sNfL levels were rather unlikely at the time of initiating the second therapy [24,25,42].

The study is limited by its non-controlled real-world setting and unknown existence of confounders in the patient cohorts. In addition, as MS is a relapsing–remitting disease, treatment effects observed in this observational setting may in part reflect natural remission following a relapse. The relatively short follow-up of at least 12 months and the small sample size in some subgroups limit outcome considerations over the long-term and warrant caution in the interpretation of the findings. Similarly, due to the retrospective approach only a limited number of sNfL and sGFAP measurements were available. Thus, subgroup analysis comparing patients with high and low sNfL or sGFAP levels was not possible.

In conclusion, the results from this retrospective data analysis demonstrate that switching highly active RMS patients from anti-CD20 therapies to cladribine and vice versa is a viable and safe treatment option.

## Author contributions

Conceptualization, FFK, TS; methodology, FFK, KFJ, SH, EW, JK; formal analysis, FFK, KFJ; data curation, FFK; writing—original draft preparation, FFK, TS; writing—review and editing, SP, KFJ, KG, BEH, KWS, SH, KB, RL, EW, MP, JK, SGM, CK, RP.

## Funding

No funding was received towards this work.

## Declaration of competing interest

The authors declare the following financial interests/personal relationships which may be considered as potential competing interests: Franz Felix Konen reports a relationship with Alexion Pharmaceuticals Inc that includes: speaking and lecture fees and travel reimbursement. Franz Felix Konen reports a relationship with argenx Germany GmbH that includes: speaking and lecture fees and travel reimbursement. Franz Felix Konen reports a relationship with Merck & Co Inc that includes: funding grants, speaking and lecture fees, and travel reimbursement. Franz Felix Konen reports a relationship with Novartis that includes: funding grants, speaking and lecture fees, and travel reimbursement. Franz Felix Konen reports a relationship with Takeda Pharmaceutical Company Limited that includes: speaking and lecture fees and travel reimbursement. Franz Felix Konen reports a relationship with German Research Foundation that includes: funding grants. Steffen Pfeuffer reports a relationship with argenx Germany GmbH that includes: consulting or advisory and speaking and lecture fees. Steffen Pfeuffer reports a relationship with Alexion Pharmaceuticals Inc that includes: consulting or advisory, speaking and lecture fees, and travel reimbursement. Steffen Pfeuffer reports a relationship with Biogen Inc that includes: consulting or advisory, funding grants, speaking and lecture fees, and travel reimbursement. Steffen Pfeuffer reports a relationship with Hexal AG that includes: consulting or advisory and speaking and lecture fees. Steffen Pfeuffer reports a relationship with Merck & Co Inc that includes: consulting or advisory, funding grants, speaking and lecture fees, and travel reimbursement. Steffen Pfeuffer reports a relationship with Novartis that includes: consulting or advisory, funding grants, speaking and lecture fees, and travel reimbursement. Steffen Pfeuffer reports a relationship with Roche that includes: consulting or advisory, speaking and lecture fees, and travel reimbursement. Steffen Pfeuffer reports a relationship with Sanofi SA that includes: consulting or advisory and speaking and lecture fees. Steffen Pfeuffer reports a relationship with Neuraxpharm Arzneimittel Gmbh that includes: travel reimbursement. Konstantin Fritz Jendretzky reports a relationship with Else Kroner-Fresenius Foundation that includes: funding grants. Konstantin Fritz Jendretzky reports a relationship with Neuraxpharm Arzneimittel Gmbh that includes: travel reimbursement. Konstantin Fritz Jendretzky reports a relationship with Merck & Co Inc that includes: travel reimbursement. Konstantin Fritz Jendretzky reports a relationship with Novartis Pharmaceuticals Corporation that includes: travel reimbursement. Klaus Gehring reports a relationship with Genzyme Corporation that includes: consulting or advisory, speaking and lecture fees, and travel reimbursement. Klaus Gehring reports a relationship with Roche that includes: consulting or advisory, speaking and lecture fees, and travel reimbursement. Klaus Gehring reports a relationship with Biogen that includes: consulting or advisory, speaking and lecture fees, and travel reimbursement. Klaus Gehring reports a relationship with Novartis that includes: consulting or advisory, speaking and lecture fees, and travel reimbursement. Birte Elias-Hamp reports a relationship with Alexion that includes: consulting or advisory, speaking and lecture fees, and travel reimbursement. Birte Elias-Hamp reports a relationship with Almirall SA that includes: consulting or advisory, speaking and lecture fees, and travel reimbursement. Birte Elias-Hamp reports a relationship with Amgen Inc that includes: consulting or advisory, speaking and lecture fees, and travel reimbursement. Birte Elias-Hamp reports a relationship with Bayer HealthCare AG that includes: consulting or advisory, speaking and lecture fees, and travel reimbursement. Birte Elias-Hamp reports a relationship with Biogen that includes: consulting or advisory, speaking and lecture fees, and travel reimbursement. Birte Elias-Hamp reports a relationship with Bristol Myers Squibb Co that includes: consulting or advisory, speaking and lecture fees, and travel reimbursement. Birte Elias-Hamp reports a relationship with FARCO-PHARMA GmbH that includes: consulting or advisory, speaking and lecture fees, and travel reimbursement. Birte Elias-Hamp reports a relationship with Hexal AG that includes: consulting or advisory, speaking and lecture fees, and travel reimbursement. Birte Elias-Hamp reports a relationship with MedDay Pharmaceuticals that includes: consulting or advisory, speaking and lecture fees, and travel reimbursement. Birte Elias-Hamp reports a relationship with Neuraxpharm Arzneimittel Gmbh that includes: consulting or advisory, speaking and lecture fees, and travel reimbursement. Birte Elias-Hamp reports a relationship with Novartis that includes: consulting or advisory, speaking and lecture fees, and travel reimbursement. Birte Elias-Hamp reports a relationship with Roche that includes: consulting or advisory, speaking and lecture fees, and travel reimbursement. Birte Elias-Hamp reports a relationship with Sanofi that includes: consulting or advisory, speaking and lecture fees, and travel reimbursement. Birte Elias-Hamp reports a relationship with Teva Pharmaceuticals USA Inc that includes: consulting or advisory, speaking and lecture fees, and travel reimbursement. Birte Elias-Hamp reports a relationship with Viatris Inc that includes: consulting or advisory, speaking and lecture fees, and travel reimbursement.

Kurt-Wolfram Suehs reports a relationship with Bavarian Nordic GmbH that includes: speaking and lecture fees and travel reimbursement. Kurt-Wolfram Suehs reports a relationship with Biogen Inc that includes: speaking and lecture fees and travel reimbursement. Kurt-Wolfram Suehs reports a relationship with Bristol-Myers Squibb Company that includes: funding grants, speaking and lecture fees, and travel reimbursement. Kurt-Wolfram Suehs reports a relationship with Merck & Co Inc that includes: speaking and lecture fees and travel reimbursement. Kurt-Wolfram Suehs reports a relationship with Mylan Pharmaceuticals Inc that includes: speaking and lecture fees and travel reimbursement. Kurt-Wolfram Suehs reports a relationship with Novartis Pharmaceuticals Corporation that includes: speaking and lecture fees and travel reimbursement. Kurt-Wolfram Suehs reports a relationship with Roche that includes: speaking and lecture fees and travel reimbursement. Kurt-Wolfram Suehs reports a relationship with Viatris Inc that includes: speaking and lecture fees and travel reimbursement. Marc Pawlitzki reports a relationship with Alexion Pharma Germany GmbH that includes: speaking and lecture fees and travel reimbursement. Marc Pawlitzki reports a relationship with argenx Germany GmbH that includes: funding grants, speaking and lecture fees, and travel reimbursement. Marc Pawlitzki reports a relationship with Bayer HealthCare AG that includes: speaking and lecture fees and travel reimbursement. Marc Pawlitzki reports a relationship with Biogen that includes: funding grants, speaking and lecture fees, and travel reimbursement. Marc Pawlitzki reports a relationship with Hexal AG that includes: funding grants, speaking and lecture fees, and travel reimbursement. Marc Pawlitzki reports a relationship with Merck & Co Inc that includes: speaking and lecture fees and travel reimbursement. Marc Pawlitzki reports a relationship with Neuraxpharm Arzneimittel Gmbh that includes: speaking and lecture fees and travel reimbursement. Marc Pawlitzki reports a relationship with Novartis Pharmaceuticals Corporation that includes: funding grants, speaking and lecture fees, and travel reimbursement. Marc Pawlitzki reports a relationship with Roche that includes: speaking and lecture fees and travel reimbursement. Marc Pawlitzki reports a relationship with Sanofi that includes: speaking and lecture fees and travel reimbursement. Marc Pawlitzki reports a relationship with Takeda Pharmaceuticals USA Inc that includes: speaking and lecture fees and travel reimbursement. Marc Pawlitzki reports a relationship with Teva Pharmaceutical Industries Ltd that includes: speaking and lecture fees and travel reimbursement. Marc Pawlitzki reports a relationship with B Braun Foundation that includes: funding grants. Marc Pawlitzki reports a relationship with German Multiple Sclerosis Society that includes: funding grants. Jens Kuhle reports a relationship with Swiss National Science Foundation that includes: funding grants. Jens Kuhle reports a relationship with Swiss Multiple Sclerosis Society that includes: funding grants. Jens Kuhle reports a relationship with Biogen Inc that includes: funding grants. Jens Kuhle reports a relationship with Celgene Corporation that includes: funding grants. Jens Kuhle reports a relationship with Merck & Co Inc that includes: funding grants. Jens Kuhle reports a relationship with Novartis Pharmaceuticals Corporation that includes: funding grants. Jens Kuhle reports a relationship with Roche that includes: funding grants. Jens Kuhle reports a relationship with Sanofi that includes: funding grants. Jens Kuhle reports a relationship with International Progressive MS Alliance that includes: funding grants. Jens Kuhle reports a relationship with University of Basel that includes: funding grants. Jens Kuhle reports a relationship with Octave Bioscience Inc that includes: funding grants. Sven G. Meuth reports a relationship with argenx Germany GmbH that includes: consulting or advisory, funding grants, speaking and lecture fees, and travel reimbursement. Sven G. Meuth reports a relationship with Alexion that includes: consulting or advisory, funding grants, speaking and lecture fees, and travel reimbursement. Sven G. Meuth reports a relationship with Almirall SA that includes: consulting or advisory, funding grants, speaking and lecture fees, and travel reimbursement. Sven G. Meuth reports a relationship with Amicus Therapeutics S.r.l. that includes: consulting or advisory, funding grants, speaking and lecture fees, and travel reimbursement. Sven G. Meuth reports a relationship with AstraZeneca Pharmaceuticals LP that includes: consulting or advisory, funding grants, speaking and lecture fees, and travel reimbursement. Sven G. Meuth reports a relationship with Bayer HealthCare AG that includes: consulting or advisory, funding grants, speaking and lecture fees, and travel reimbursement. Sven G. Meuth reports a relationship with Biogen that includes: consulting or advisory, funding grants, speaking and lecture fees, and travel reimbursement. Sven G. Meuth reports a relationship with BioNTech SE that includes: consulting or advisory, funding grants, speaking and lecture fees, and travel reimbursement. Sven G. Meuth reports a relationship with Bristol Myers Squibb Co that includes: consulting or advisory, funding grants, speaking and lecture fees, and travel reimbursement. Sven G. Meuth reports a relationship with Celgene Corporation that includes: consulting or advisory, funding grants, speaking and lecture fees, and travel reimbursement. Sven G. Meuth reports a relationship with Datamed Ltd that includes: consulting or advisory, funding grants, speaking and lecture fees, and travel reimbursement. Sven G. Meuth reports a relationship with Desitin Pharmaceuticals that includes: consulting or advisory, funding grants, speaking and lecture fees, and travel reimbursement. Sven G. Meuth reports a relationship with Diamed-Pharma LTD that includes: consulting or advisory, funding grants, speaking and lecture fees, and travel reimbursement. Sven G. Meuth reports a relationship with Dresden International University GmbH that includes: consulting or advisory, funding grants, speaking and lecture fees, and travel reimbursement. Sven G. Meuth reports a relationship with Genzyme Corporation that includes: consulting or advisory, funding grants, speaking and lecture fees, and travel reimbursement. Sven G. Meuth reports a relationship with Hexal AG that includes: consulting or advisory, funding grants, speaking and lecture fees, and travel reimbursement. Sven G. Meuth reports a relationship with Janssen-Cilag Ltd that includes: consulting or advisory, funding grants, speaking and lecture fees, and travel reimbursement. Sven G. Meuth reports a relationship with MedDay Pharmaceuticals that includes: consulting or advisory, funding grants, speaking and lecture fees, and travel reimbursement. Sven G. Meuth reports a relationship with Merck & Co Inc that includes: consulting or advisory, funding grants, speaking and lecture fees, and travel reimbursement. Sven G. Meuth reports a relationship with Mylan Pharmaceuticals Inc that includes: consulting or advisory, funding grants, speaking and lecture fees, and travel reimbursement. Sven G. Meuth reports a relationship with Neuraxpharm Arzneimittel Gmbh that includes: consulting or advisory, funding grants, speaking and lecture fees, and travel reimbursement. Sven G. Meuth reports a relationship with Novartis Pharmaceuticals Corporation that includes: consulting or advisory, funding grants, speaking and lecture fees, and travel reimbursement. Sven G. Meuth reports a relationship with Novo Nordisk Inc that includes: consulting or advisory, funding grants, speaking and lecture fees, and travel reimbursement. Sven G. Meuth reports a relationship with Ono Pharmaceutical Co Ltd that includes: consulting or advisory, funding grants, speaking and lecture fees, and travel reimbursement. Sven G. Meuth reports a relationship with Oxford PharmaGenesis Ltd that includes: consulting or advisory, funding grants, speaking and lecture fees, and travel reimbursement. Sven G. Meuth reports a relationship with Quintiles that includes: consulting or advisory, funding grants, speaking and lecture fees, and travel reimbursement. Sven G. Meuth reports a relationship with Roche that includes: consulting or advisory, funding grants, speaking and lecture fees, and travel reimbursement. Sven G. Meuth reports a relationship with Sanofi that includes: consulting or advisory, funding grants, speaking and lecture fees, and travel reimbursement. Sven G. Meuth reports a relationship with Springer Medizin Verlag GmbH that includes: consulting or advisory, funding grants, speaking and lecture fees, and travel reimbursement. Sven G. Meuth reports a relationship with STADA Arzneimittel AG that includes: consulting or advisory, funding grants, speaking and lecture fees, and travel reimbursement. Sven G. Meuth reports a relationship with Chugai Pharmaceutical Co Ltd that includes: consulting or advisory, funding grants, speaking and lecture fees, and travel reimbursement. Sven G. Meuth reports a relationship with Teva Pharmaceutical Industries Ltd that includes: consulting or advisory, funding grants, speaking and lecture fees, and travel reimbursement. Sven G. Meuth reports a relationship with Viatris Inc that includes: consulting or advisory, funding grants, speaking and lecture fees, and travel reimbursement. Sven G. Meuth reports a relationship with Xcenda GmbH that includes: consulting or advisory, funding grants, speaking and lecture fees, and travel reimbursement. Sven G. Meuth reports a relationship with Federal Ministry of Education and Research Berlin Office that includes: funding grants. Sven G. Meuth reports a relationship with German Federal Institute for Risk Assessment that includes: funding grants. Sven G. Meuth reports a relationship with German Research Foundation that includes: funding grants. Sven G. Meuth reports a relationship with Else Kroner-Fresenius Foundation that includes: funding grants. Sven G. Meuth reports a relationship with German Academic Exchange Service that includes: funding grants. Sven G. Meuth reports a relationship with Hertie Foundation that includes: funding grants. Sven G. Meuth reports a relationship with German Neurology Foundation that includes: funding grants. Sven G. Meuth reports a relationship with Ministry of Culture and Science of the State of North Rhine-Westphalia that includes: funding grants. Sven G. Meuth reports a relationship with Daimler and Benz Foundation that includes: funding grants. Sven G. Meuth reports a relationship with German Multiple Sclerosis Society that includes: funding grants. Sven G. Meuth reports a relationship with Hempel Foundation that includes: funding grants. Sven G. Meuth reports a relationship with German Alzheimer Society that includes: funding grants. Sven G. Meuth reports a relationship with Fresenius Medical Care AG that includes: funding grants. Christoph Kleinschnitz reports a relationship with Alexion Pharma Germany GmbH that includes: speaking and lecture fees and travel reimbursement. Christoph Kleinschnitz reports a relationship with Almirall SA that includes: speaking and lecture fees and travel reimbursement. Christoph Kleinschnitz reports a relationship with AstraZeneca Pharmaceuticals LP that includes: speaking and lecture fees and travel reimbursement. Christoph Kleinschnitz reports a relationship with Bayer Corporation that includes: speaking and lecture fees and travel reimbursement. Christoph Kleinschnitz reports a relationship with Biogen Inc that includes: speaking and lecture fees and travel reimbursement. Christoph Kleinschnitz reports a relationship with BioNTech SE that includes: speaking and lecture fees and travel reimbursement. Christoph Kleinschnitz reports a relationship with Boehringer Ingelheim Pharma GmbH & Co KG that includes: speaking and lecture fees and travel reimbursement. Christoph Kleinschnitz reports a relationship with Bristol-Myers Squibb Company that includes: speaking and lecture fees and travel reimbursement. Christoph Kleinschnitz reports a relationship with Daiichi Sankyo Inc that includes: speaking and lecture fees and travel reimbursement. Christoph Kleinschnitz reports a relationship with Merck & Co Inc that includes: speaking and lecture fees and travel reimbursement. Christoph Kleinschnitz reports a relationship with Mylan Pharmaceuticals Inc that includes: speaking and lecture fees and travel reimbursement. Christoph Kleinschnitz reports a relationship with Novartis that includes: speaking and lecture fees and travel reimbursement. Christoph Kleinschnitz reports a relationship with Pfizer Inc that includes: speaking and lecture fees and travel reimbursement. Christoph Kleinschnitz reports a relationship with Roche that includes: speaking and lecture fees and travel reimbursement. Christoph Kleinschnitz reports a relationship with Sanofi that includes: speaking and lecture fees and travel reimbursement. Christoph Kleinschnitz reports a relationship with STADA Arzneimittel AG that includes: speaking and lecture fees and travel reimbursement. Christoph Kleinschnitz reports a relationship with Teva Pharmaceutical Industries Ltd that includes: speaking and lecture fees and travel reimbursement. Christoph Kleinschnitz reports a relationship with Janssen-Cilag Ltd that includes: speaking and lecture fees and travel reimbursement. Christoph Kleinschnitz reports a relationship with Horizon Therapeutics USA Inc that includes: speaking and lecture fees and travel reimbursement. Christoph Kleinschnitz reports a relationship with Medscape LLC that includes: speaking and lecture fees and travel reimbursement. Christoph Kleinschnitz reports a relationship with Hexal AG that includes: speaking and lecture fees and travel reimbursement. Refik Pul reports a relationship with Biogen that includes: speaking and lecture fees and travel reimbursement. Refik Pul reports a relationship with Bristol Myers Squibb Co that includes: speaking and lecture fees and travel reimbursement. Refik Pul reports a relationship with Horizon Therapeutics USA Inc that includes: speaking and lecture fees and travel reimbursement. Refik Pul reports a relationship with Janssen Pharmaceuticals Inc that includes: speaking and lecture fees and travel reimbursement. Refik Pul reports a relationship with Merck & Co Inc that includes: funding grants, speaking and lecture fees, and travel reimbursement. Refik Pul reports a relationship with Novartis that includes: funding grants, speaking and lecture fees, and travel reimbursement. Refik Pul reports a relationship with Roche that includes: speaking and lecture fees and travel reimbursement. Refik Pul reports a relationship with Sanofi that includes: speaking and lecture fees and travel reimbursement. Thomas Skripuletz reports a relationship with Alnylam Pharmaceuticals Inc that includes: consulting or advisory, funding grants, speaking and lecture fees, and travel reimbursement. Thomas Skripuletz reports a relationship with CSL Behring GmbH that includes: funding grants, speaking and lecture fees, and travel reimbursement. Thomas Skripuletz reports a relationship with Merck & Co Inc that includes: consulting or advisory, funding grants, speaking and lecture fees, and travel reimbursement. Thomas Skripuletz reports a relationship with Novartis that includes: consulting or advisory, funding grants, speaking and lecture fees, and travel reimbursement. Thomas Skripuletz reports a relationship with Siemens that includes: funding grants, speaking and lecture fees, and travel reimbursement. Thomas Skripuletz reports a relationship with Alexion Pharmaceuticals Inc that includes: consulting or advisory, speaking and lecture fees, and travel reimbursement. Thomas Skripuletz reports a relationship with Amgen Inc that includes: consulting or advisory, speaking and lecture fees, and travel reimbursement. Thomas Skripuletz reports a relationship with argenx Germany GmbH that includes: consulting or advisory, speaking and lecture fees, and travel reimbursement. Thomas Skripuletz reports a relationship with Bayer Corporation that includes: consulting or advisory, speaking and lecture fees, and travel reimbursement. Thomas Skripuletz reports a relationship with Biogen Inc that includes: consulting or advisory, speaking and lecture fees, and travel reimbursement. Thomas Skripuletz reports a relationship with Bristol Myers Squibb Co that includes: consulting or advisory, speaking and lecture fees, and travel reimbursement. Thomas Skripuletz reports a relationship with Centogene GmbH that includes: consulting or advisory, speaking and lecture fees, and travel reimbursement. Thomas Skripuletz reports a relationship with Grifols Inc that includes: consulting or advisory, speaking and lecture fees, and travel reimbursement. Thomas Skripuletz reports a relationship with Hexal AG that includes: consulting or advisory, speaking and lecture fees, and travel reimbursement. Thomas Skripuletz reports a relationship with Horizon Therapeutics USA Inc that includes: consulting or advisory, speaking and lecture fees, and travel reimbursement. Thomas Skripuletz reports a relationship with Janssen Pharmaceuticals Inc that includes: consulting or advisory, speaking and lecture fees, and travel reimbursement. Thomas Skripuletz reports a relationship with Pfizer Inc that includes: consulting or advisory, speaking and lecture fees, and travel reimbursement. Thomas Skripuletz reports a relationship with Roche that includes: consulting or advisory, speaking and lecture fees, and travel reimbursement. Thomas Skripuletz reports a relationship with Sanofi that includes: consulting or advisory, speaking and lecture fees, and travel reimbursement. Thomas Skripuletz reports a relationship with Sobi Inc that includes: consulting or advisory, speaking and lecture fees, and travel reimbursement. Thomas Skripuletz reports a relationship with Teva Pharmaceuticals USA Inc that includes: consulting or advisory, speaking and lecture fees, and travel reimbursement. Thomas Skripuletz reports a relationship with Viatris that includes: consulting or advisory, speaking and lecture fees, and travel reimbursement. If there are other authors, they declare that they have no known competing financial interests or personal relationships that could have appeared to influence the work reported in this paper.
