# Supplementary material for: Switching from anti-CD20 therapies to cladribine and vice versa – Analysis of a German relapsing multiple sclerosis cohort
Source: Neurotherapeutics. 2025 Dec 4;23(1):e00812. doi: 10.1016/j.neurot.2025.e00812 (PMC12976515; doi:10.1016/j.neurot.2025.e00812)
Supplement: Multimedia component 1 [file mmc1.docx]

**Supplementary Table S1** Baseline characteristics, stratified by reason for switch

| **First study treatment** | **Anti-CD20** | | | **Cladribine** | | |
| --- | --- | --- | --- | --- | --- | --- |
| **Switch reason** | **Disease activity (N=22)** | **Adverse events (N=9)** | **p-value** | **Disease activity (N=34)** | **Adverse events (N=6)** | **p-value** |
| Age, years, median (IQR) | 43  (34-52) | 48  (37-56) | 0.4336 | 37  (27-42) | 38.5  (33-43) | 0.6637 |
| Females, n (%) | 17  (77%) | 5  (56%) | 0.3846 | 24  (71%) | 5  (83%) | 0.9999 |
| EDSS, median (IQR) | 4  (3-6) | 3  (2-6) | 0.3261 | 2.5  (2-4) | 3  (2-4) | 0.6562 |
| Previous therapies, median number (IQR) | 3  (2-5) | 2  (1-3) | 0.3337 | 2  (2-4) | 2  (1-5) | 0.8628 |
| Therapy naïve, n (%) | 2  (9%) | 2  (22%) | 0.5595 | 8  (24%) | 2  (33%) | 0.6287 |
| Months from first MS diagnosis to first therapy, median (IQR) | 49  (17-189) | 124  (57-253) | 0.1849 | 46  (11-110) | 28  (1-77) | 0.1866 |
| Treatment courses first therapy, median (IQR)^a^ | 3  (2-5) | 4  (2-4) | 0.6368 | 2  (2-4) | 2.5  (2-4) | 0.7283 |
| Days between therapies, median (IQR) | 211  (155-271) | 290  (184-574) | 0.2540 | 547  (335-923) | 479  (371-559) | 0.7284 |
| Reasons for treatment switch  Relapse  PIRA  MRI activity  ADR Lymphopenia | 12 (55 %)  5 (23 %)  5 (23 %) | 9 (100%)  0 |  | 19 (56%)  1 (3%)  14 (41%) | 6 (100%)  6 (100%) |  |

^a^For cladribine: one course = 1 treatment week; for ocrelizumab: one course = 600 mg

EDSS = Expanded Disability Status Scale, IQR = interquartile range; PIRA = progression independent of relapse activity; ADR = adverse drug reaction
