# Supplementary material for: Switching from anti-CD20 therapies to cladribine and vice versa – Analysis of a German relapsing multiple sclerosis cohort
Source: Neurotherapeutics. 2025 Dec 4;23(1):e00812. doi: 10.1016/j.neurot.2025.e00812 (PMC12976515; doi:10.1016/j.neurot.2025.e00812)
Supplement: Multimedia component 2 [file mmc2.docx]

**Supplementary Table S2** Baseline characteristics, stratified by age at initiation of first study treatment

| **First study treatment** | **Anti-CD20** | | | **Cladribine** | | |
| --- | --- | --- | --- | --- | --- | --- |
| **Age at initiation** | **<45 years**  **(N=16)** | **≥45 years**  **(N=15)** | **p-value** | **<45 years**  **(N=32)** | **≥45 years**  **(N=8)** | **p-value** |
| Age, years, median (IQR) | 34  (29-42) | 53  (50-58) | <0.0001 | 33  (26-40) | 47.5  (47-51) | <0.0001 |
| Females, n (%) | 12  (75%) | 10  (67%) | 0.7043 | 22  (69%) | 7  (88%) | 0.4055 |
| EDSS, median (IQR) | 4  (3-6) | 4  (3-6) | 0.2745 | 2  (1.5-3) | 4.5  (3-5.5) | 0.0121 |
| Previous therapies, median number (IQR) | 4  (2-5.5) | 2  (1-5) | 0.7962 | 2  (2-4) | 2  (1-3) | 0.3249 |
| Therapy naïve, n (%) | 2  (13%) | 2  (13%) | 0.9999 | 9  (28%) | 1  (13%) | 0.6526 |
| Months from first MS diagnosis to first therapy, median (IQR) | 58  (21-225) | 167  (26-280) | 0.2127 | 49  (7-106) | 38  (8-107) | 0.9274 |
| Treatment courses first therapy, median (IQR)^a^ | 3  (2-4) | 4  (3-5) | 0.6768 | 2  (2-4) | 4  (2-4) | 0.2914 |
| Days between therapies, median (IQR) | 211  (156-261) | 243  (181-303) | 0.5196 | 453  (350-861) | 563  (383-1124) | 0.3024 |
| Reasons for treatment switch  Relapse  PIRA  MRI activity  ADR | 8 (50 %)  1 (6 %)  4 (25 %)  3 (19 %) | 4 (26.7%)  4 (26.7%)  1 (6.7%)  6 (40%) | 0.2734  0.1719  0.3326  0.2524 | 14 (44%)  0 (0%)  13 (41%)  5 (15%) | 5 (62.5%)  1 (12.5%)  1 (12.5%)  1 (12.5%) | 0.4420  0.2000  0.2216  0.9999 |

^a^For cladribine: one course = 1 treatment week; for ocrelizumab: one course = 600 mg

EDSS = Expanded Disability Status Scale, IQR = interquartile range; PIRA = progression independent of relapse activity; ADR = adverse drug reaction
